# Association between DNA haplogroups and severe sepsis in patients who underwent major surgery

**DOI:** 10.1186/cc13414

**Published:** 2014-03-17

**Authors:** E Gomez, M Heredia, S Resino, M Jimenez-Sousa, J Gomez-Herreras, E Tamayo, P Jorge, M Lorenzo, P Ruiz

**Affiliations:** 1 Hospital Clínico Universitario de Valladolid, Spain; 2 Instituto de Salud Carlos III, Madrid, Spain; 3 Hospital Río Hortega, Valladolid, Spain

## Introduction

The aim of this study was to analyze whether mitochondrial DNA haplogroups are associated with severe sepsis and mortality after major surgery in European populations. Mitochondrial DNA (mtDNA) variants may play an important role for predicting clinical outcome in sepsis [1].

## Methods

We carried out a case-control study on 240 septic patients (severe sepsisor septic shock; Case group) and 267 patients with systemic inflammatory response syndrome (SIRS; Control group). Furthermore, a longitudinal substudy for analyzing survival was performed in septic patients. mtDNA genotyping wasperformed by Sequenom's MassARRAY platform.

## Results

Regarding cardiac surgery, patients with clusters JT and haplogroup J hadhigher likelihoods of sepsis than patients with clusters HV (OR = 2.76 (95% CI = 1.27; 6.02); *P *= 0.010) and haplogroup H (OR = 3.68 (95% CI = 1.17; 11.54); *P *= 0.026), respectively. No significant association was found for abdominal surgery. When analyzing survival, 45.4% patients died with a survival median of 39 (95% CI = 31.4; 46.62) days. When the clusters were analyzed for all patients, 41% (55/134) of patients within cluster HV died versus 71.4% (10/14) patients within cluster IWX (*P *= 0.018). The adjusted Cox regression showed that patients within cluster IWX had a higher risk of dying than patients within cluster HV (hazard ratio (HR) = 2.24 (95% CI = 1.10; 4.56); *P *= 0.027). No significant association between haplogroups and mortality was found when patients were stratified by the type of surgery.

## Conclusion

European mitochondrial haplogroups are associated with sepsis developmentin patients who underwent major cardiac surgery, but not in patients who underwent major abdominal surgery. Besides, mtDNA haplogroups also influence mortality. Haplogroups HV and H were related to low risk of sepsis and death, while JT and J were related to high risk of sepsis, and IWX was associated with death.
